# Assessment of myocardial fibrosis in patients with systemic sclerosis using [^68^Ga]Ga-FAPI-04-PET-CT

**DOI:** 10.1007/s00259-022-06081-4

**Published:** 2022-12-16

**Authors:** Christoph Treutlein, Jörg H. W. Distler, Koray Tascilar, Sara Chenguiti Fakhouri, Andrea-Hermina Györfi, Armin Atzinger, Alexandru-Emil Matei, Clara Dees, Maike Büttner-Herold, Torsten Kuwert, Olaf Prante, Tobias Bäuerle, Michael Uder, Georg Schett, Christian Schmidkonz, Christina Bergmann

**Affiliations:** 1 Department of Radiology, Friedrich-Alexander University Erlangen-Nürnberg (FAU) and Universitätsklinikum Erlangen, Erlangen, Germany; 2 Department of Internal Medicine 3–Rheumatology and Immunology, FAU Erlangen-Nürnberg and Universitätsklinikum Erlangen, Erlangen, Germany; 3 Deutsches Zentrum Immuntherapie (DZI), FAU Erlangen-Nürnberg and Universitätsklinikum Erlangen, Erlangen, Germany; 4 Department of Nuclear Medicine, FAU Erlangen-Nürnberg and Universitätsklinikum Erlangen, Erlangen, Germany; 5 Department of Nephropathology, Institute of Pathology, Friedrich-Alexander-Universität Erlangen-Nürnberg (FAU), Erlangen, Germany; 6 University of Applied Sciences Amberg-Weiden, Institute for Medical Engineering, 92637 Weiden, Germany

**Keywords:** Myocardial fibrosis, Systemic sclerosis, [^68^Ga]Ga-FAPI-04-PET-CT, Cardiac MRI

## Abstract

**Purpose:**

Myocardial fibrosis (MF) is a factor of poor prognosis in systemic sclerosis (SSc). Direct *in-vivo* visualization of fibroblast activation as early readout of MF has not been feasible to date. Here, we characterize ^68^Gallium-labeled-Fibroblast-Activation-Inhibitor-04 ([^68^Ga]Ga-FAPI-04)-PET-CT as a diagnostic tool in SSc-related MF.

**Methods:**

In this proof-of-concept trial, six SSc patients with and eight without MF of the EUSTAR cohort Erlangen underwent [^68^Ga]Ga-FAPI-04-PET-CT and cardiac MRI (cMRI) and clinical and serologic investigations just before baseline and during follow-up between January 2020 and December 2020. Myocardial biopsy was performed as clinically indicated.

**Results:**

[^68^Ga]Ga-FAPI-04 tracer uptake was increased in SSc-related MF with higher uptake in SSc patients with arrhythmias, elevated serum-NT-pro-BNP, and increased late gadolinium enhancement (LGE) in cMRI. Histologically, myocardial biopsies from cMRI- and [^68^Ga]Ga-FAPI-04-positive regions confirmed the accumulation of FAP^+^ fibroblasts surrounded by collagen deposits. We observed similar but not equal spatial distributions of [^68^Ga]Ga-FAPI-04 uptake and quantitative cMRI-based techniques. Using sequential [^68^Ga]Ga-FAPI-04-PET-CTs, we observed dynamic changes of [^68^Ga]Ga-FAPI-04 uptake associated with changes in the activity of SSc-related MF, while cMRI parameters remained stable after regression of molecular activity and rather indicated tissue damage.

**Conclusions:**

We present first in-human evidence that [^68^Ga]Ga-FAPI-04 uptake visualizes fibroblast activation in SSc-related MF and may be a diagnostic option to monitor cardiac fibroblast activity in situ.

**Supplementary Information:**

The online version contains supplementary material available at 10.1007/s00259-022-06081-4.

## Introduction

Myocardial fibrosis occurs in response to a variety of triggers and in systemic diseases. Systemic sclerosis (SSc) is a prototypical fibrosing disorder with a high case-related mortality[1, 2]. Myocardial fibrosis (MF) is a factor of poor prognosis and a major cause of SSc-related deaths[3, 4]. Early vascular lesions with repetitive impairment of the myocardial microcirculation are thought to trigger diffuse MF [5]. Histologically, MF is characterized by the accumulation of fibroblasts and myofibroblasts [5]; the latter express alpha-smooth-muscle actin (αSMA) and secrete abundant collagen [6]. Moreover, fibroblast activation prompts the upregulation of fibroblast activation protein (FAP) [7, 8]. Exaggerated fibroblast activation results in progressive accumulation of collagen and disrupts the myocardial tissue structure. Consequently, alterations of the conduction system occur, and cardiac contractility is impaired [9]. The diagnosis and management of patients with SSc-related MF is challenging: Current diagnostics to screen SSc patients for MF including echocardiography, electrocardiogram (ECG), and serum levels of N-terminal-probrain-natriuretic-peptide (Nt-pro-BNP) are not specific for MF and not sensitive for early changes [3, 9, 10]. Endomyocardial biopsy carries an interventional risk and the risk of sampling bias [11]. Cardiac MRI (cMRI) is considered the gold standard for non-invasive diagnostics of MF [9, 11]; however, current imaging techniques including cMRI predominately monitor the accrual of fibrotic tissue damage rather than the activity of the fibrotic remodeling process. The direct, noninvasive monitoring of the current molecular activity of fibrotic remodeling in the heart has not been feasible so far.

Radiolabeled quinoline-based FAP inhibitors (FAPIs) that selectively bind FAP have recently been developed as PET tracers. The expression of FAP on cancer associated fibroblasts (CAFs) is well described, and the first in-human studies demonstrated increased FAPI uptake specifically in the tumor stroma of different malignancies [12–15]. Moreover, FAPI uptake has been characterized in autoimmune diseases including SSc-related pulmonary fibrosis [16], IgG4-related disease [17], and rheumatoid arthritis [18] in alternatively: in pilot studies. Moreover, increased myocardial uptake of a FAP targeting tracer in response to myocardial infarction has recently been described in murine models [19].

Here, we tested the hypotheses that [^68^Ga]Ga-FAPI-04 uptake can differentiate SSc patients with MF from SSc patients without MF, that increased [^68^Ga]Ga-FAPI-04 uptake is associated with unfavorable prognostic factors in SSc-MF and that [^68^Ga]Ga-FAPI-04 uptake assesses current molecular fibroblast activity rather than accumulating disease damage.

## Materials and methods

The materials and methods are described in the supplementary material.

## Results

### Accumulation of [^68^Ga]Ga-FAPI-04 in myocardial fibrosis in SSc patients

**Fig. 1:**
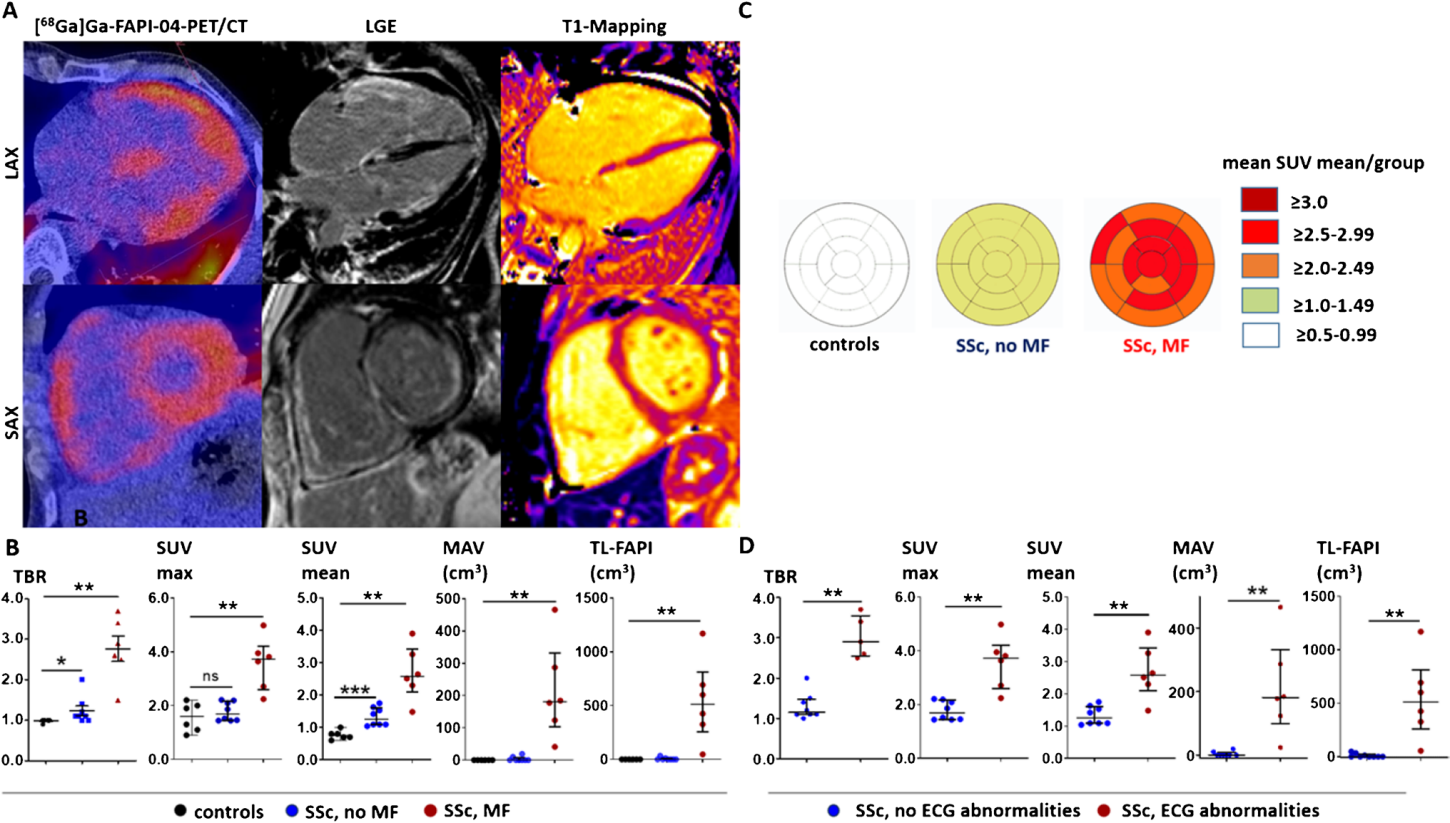
[^68^Ga]Ga-FAPI-04 uptake is increased in SSc patients with myocardial fibrosis compared to SSc patients without myocardial fibrosis and nondiseased controls. **A** Representative image of a [^68^Ga]Ga-FAPI-04-PET/CT scan of a patient with Systemic Sclerosis (SSc)-related myocardial fibrosis (MF) with tracer uptake in fibrotic areas of the myocardium of both ventricles. The corresponding cMRI confirms that [^68^Ga]Ga-FAPI-04 tracer uptake projects to areas of fibrotic tissue remodeling as visualized by late gadolinium enhancement (LGE) and prolonged T1-relaxation time*.* **B** Tissue to background ratio (TBR), maximal and mean standardized uptake values (SUV max and SUV mean (median/IQR)), metabolically active volume (MAV, cm^3^, (median/IQR)), and total lesion FAPI (TL-FAPI, cm^3^ (median/IQR)) in patients with SSc-associated MF (*n* = 6), in SSc patients without MF (*n* = 8), and in nondiseased controls (*n* = 5). **C** Mapping of the SUV mean values to the 17-regions of the AHA-model in patients with SSc-associated MF (*n* = 6), in SSc patients without MF (*n* = 8) and in nondiseased controls. Visualization of the mean SUVmean values per group in every segment. The individual distribution of SUVmean values of all SSc patients are shown in supplementary Fig. 2. **D** TBR, SUV max, SUV mean, MAV (cm^3^), and TL-FAPI (cm^3^) of SSc patients with and without ECG-abnormalities (6/8 per group corresponding to the groups with myocardial involvement). Data are presented as the median with an interquartile range. *p < 0.05; **p < 0.01; ***p < 0.001. SAX: short axis view, LAX: long axis view, ECG: electrocardiogram, myocard. dis.: myocardial disease

The baseline characteristics of all participants are shown in supplementary table 2. First, we compared myocardial [^68^Ga]Ga-FAPI-04 uptake as determined by tissue to background ratio (TBR), mean and maximal standardized uptake value (SUV mean and SUV max), metabolically active volume (MAV), and total lesion FAPI (TL-FAPI) in SSc patients with MF (SSc-MF group) to SSc patients without MF (SSc-noMF group) and to nondiseased controls (Fig. 1A, B): [^68^Ga]Ga-FAPI-04 uptake was significantly increased in the SSc-MF group compared to the SSc-noMF group and to controls (Fig. 1B). Subanalyses revealed that SSc patients without myocardial involvement on cMRI also showed numerically higher TBR and SUV mean values compared to non-SSc controls, suggesting an increased basal activation level of myocardial fibroblasts in SSc (Fig. 1B, C). The distribution of [^68^Ga]Ga-FAPI-04 uptake in the SSc-MF patients was diffuse (Fig. 1C, supplementary Fig. 2) and did not correspond to the coronary artery supply areas.

Cardiac arrhythmias and elevated serum NT-pro-BNP levels are associated with an unfavorable prognosis in SSc [3, 4, 20]. We observed increased [^68^Ga]Ga-FAPI-04 uptake values in patients with ECG-abnormalities and cardiac arrhythmias (Fig. 1D). We also observed increased [^68^Ga]Ga-FAPI-04 uptake in patients with elevated Nt-pro-BNP values (supplementary Fig. 3).

### Accumulation of FAP^+^ fibroblasts and collagen in myocardial tissue sections from myocardial areas with [^68^Ga]Ga-FAPI-04 uptake

**Fig. 2:**
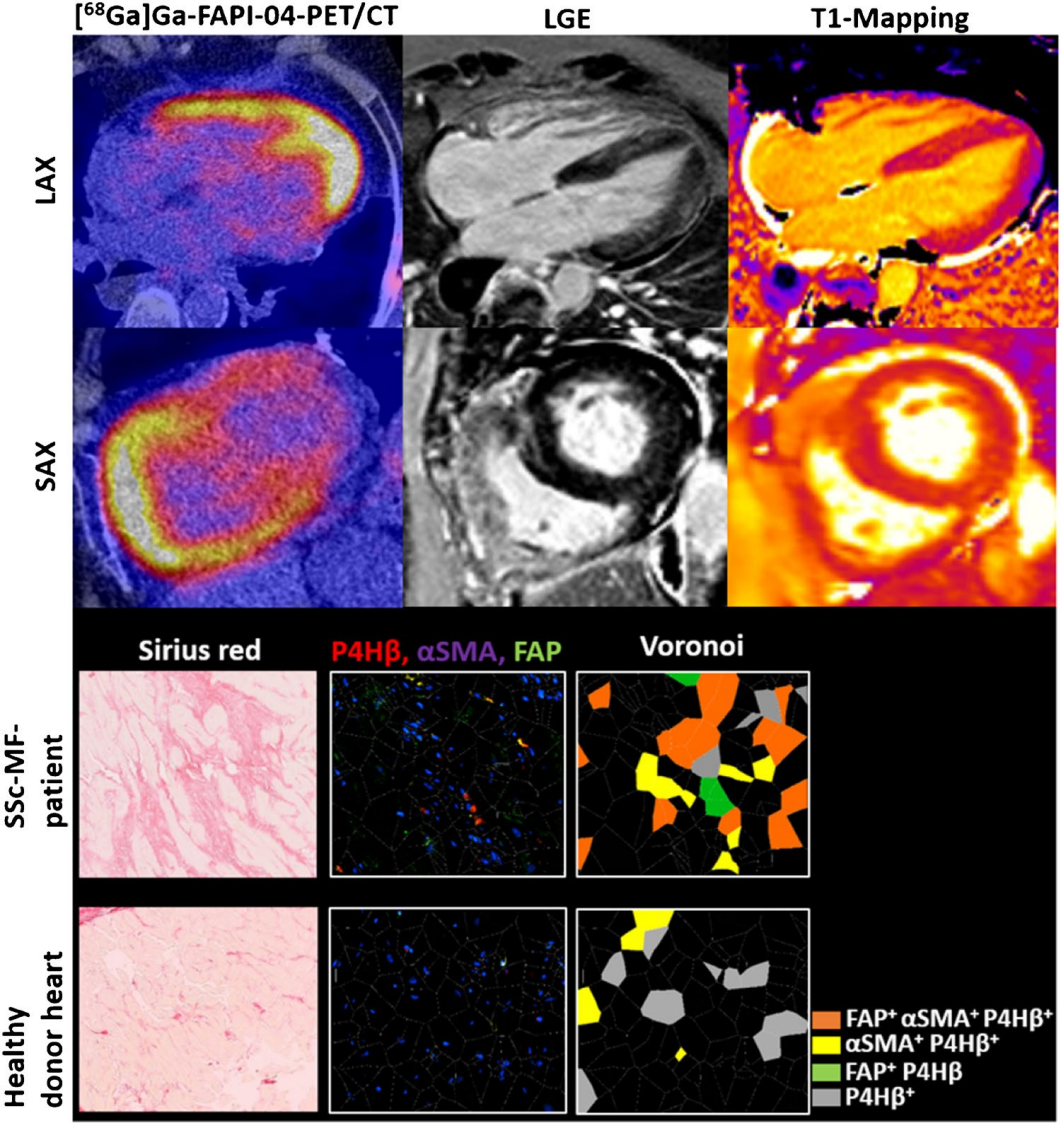
Increased [^68^Ga]Ga-FAPI-04 uptake corresponds to the accumulation of FAP^+^-expressing myofibroblasts in myocardial biopsy. Representative image of a [^68^Ga]Ga-FAPI-04-PET-CT scan and the corresponding image of cardiac MRI with late gadolinium enhancement (LGE) and T1-mapping of a patient with myocardial fibrosis (MF) in the upper part of the image. Myocardial biopsy was performed from a [^68^Ga]Ga-FAPI-04 positive- and LGE-positive region. Representative pictures of sirius red staining and immunofluorescence staining with the fibroblast marker prolyl-hydroxylase-4β (P4Hβ), the myofibroblast marker α-smooth-muscle actin (αSMA), and fibroblast activation protein (FAP) and Voronoi tessellations are shown in the lower part of the figure. Myocardial tissue sections of two healthy donor hearts of heart-transplanted patients were analyzed as controls. SAX: short axis view, LAX: long axis view

Next, we correlated the imaging findings of cMRI and [^68^Ga]Ga-FAPI-04-PET/CT with histological findings by analyzing SSc-MF myocardial biopsy samples taken from myocardial regions with increased [^68^Ga]Ga-FAPI-04 uptake and LGE in an SSc-MF patient. Therefore, image-guided collection of myocardial biopsies was performed. Myocardial biopsies of two heart-transplanted patients with healthy donor hearts were analyzed as controls. To analyze both the deposition of collagen and the number of activated fibroblasts and myofibroblasts, we performed sirius red staining and immunofluorescence staining of the pan-fibroblast marker prolyl-4-hydroxylase β(P4Hβ), αSMA, and FAP in consecutive tissue sections. We observed pronounced interstitial collagen deposition in SSc-MF compared to controls (Fig. 2). We observed higher numbers of fibroblasts (P4Hβ^+^ cells) and in particular accumulation of myofibroblasts (αSMA^+^; P4Hβ^+^) in the SSc-MF patient compared to controls. Of note, two thirds of myofibroblasts in SSc-MF stained positive for FAP.

### Comparison of [^68^Ga]Ga-FAPI-04 uptake with cMRI-based imaging and clinical parameters at baseline and follow-up

We mapped the intensity of [^68^Ga]Ga-FAPI-04 uptake (SUVmean) to the 17 regions described by the American Heart Association (AHA) and compared them to LGE and T1-mapping, respectively. Although the spatial distribution of enhanced T1-relaxation times, LGE, and [^68^Ga]Ga-FAPI-04 uptake overlapped in certain areas, they differed in others, suggesting that [^68^Ga]Ga-FAPI-PET-CT and cMRI visualize different aspects of the disease process (supplementary Figs. 4 and 5).

To further confirm that [^68^Ga]Ga-FAPI uptake assesses current molecular fibroblast activity, we analyzed associations of [^68^Ga]Ga-FAPI-04 uptake at baseline with changes of clinical parameters of SSc-MF on follow-up: left ventricular contractility (EF), changes of ECG morphology, and serum Nt-pro-BNP levels in all patients available for follow-up:

#### Early monitoring of treatment response

**Fig. 3:**
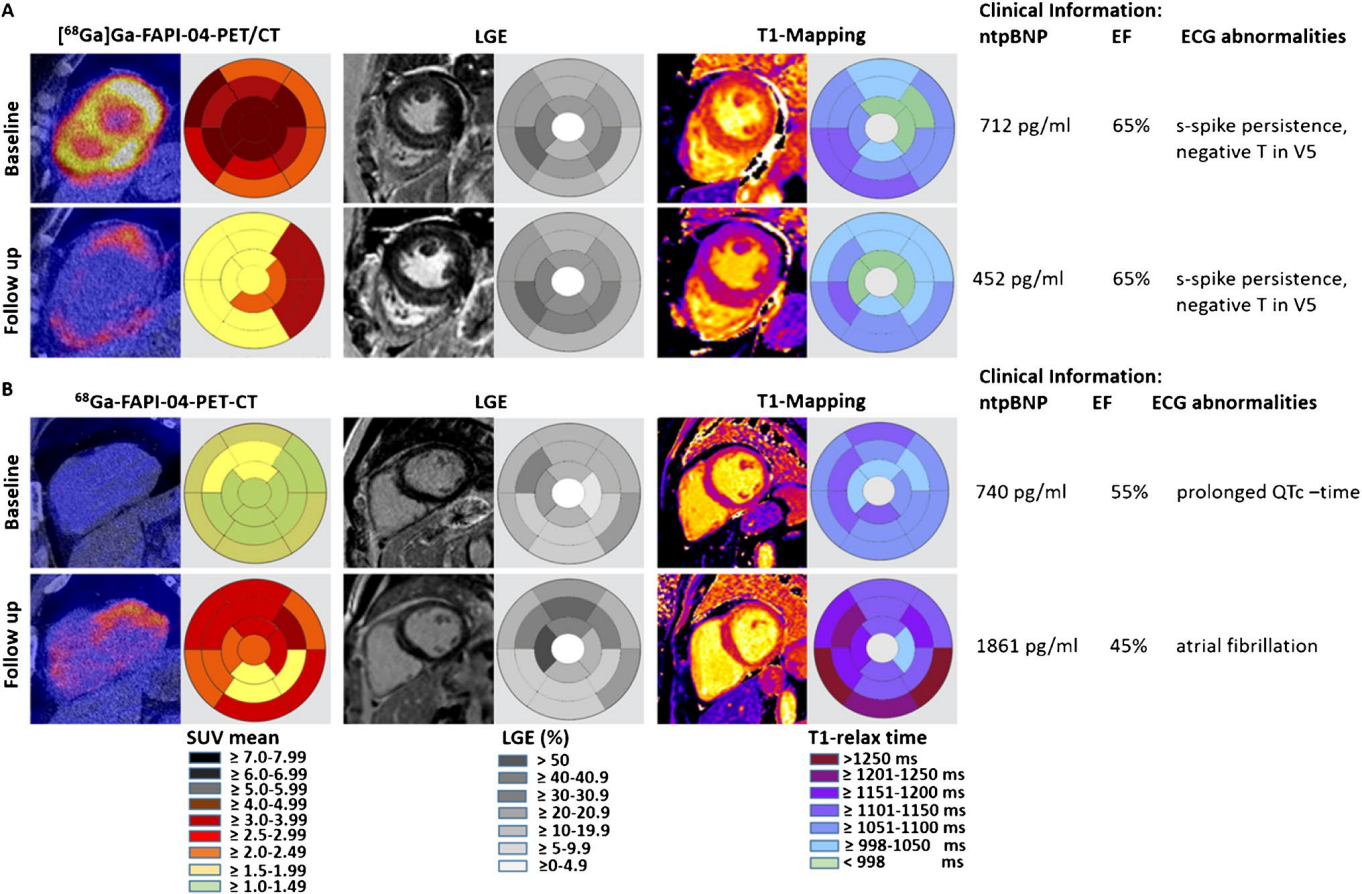
Follow-up observations of SSc-MF patients. Representative images of [^68^Ga]Ga-FAPI-04-PET-CT scan and the corresponding cMRI with late gadolinium enhancement (LGE) and T-mapping at baseline (BL) and follow-up (FU). Clinical findings including serum Nt-pro-BNP levels, ejection fraction (EF, %), and ECG findings are tabulated. **A** Reduction of [^68^Ga]Ga-FAPI-tracer uptake upon start of medical therapy in a patient with pronounced tracer uptake at baseline, stable LGE, and minor changes of T1 relaxation times (participant 13). **B** Progressive [^68^Ga]Ga-FAPI-04 tracer uptake and clinical deterioration with increased NT-pro-BNP levels and new onset of atrial fibrillation on follow op despite therapy (participant 10). The follow-up investigations of participants 11 and 14 are visualized in supplementary Fig. 6

In participant 13, who showed high myocardial [^68^Ga]Ga-FAPI-04 uptake at baseline, a marked reduction of [^68^Ga]Ga-FAPI-04 uptake occurred after initiation of mycophenolate mofetil (MMF) treatment and this was paralleled by reduction of serum Nt-pro-BNP (Fig. 3A). In contrast, LGE and EF remained stable; T1-relaxation times showed minor changes.

#### Early monitoring of MF exacerbation

Participant 10 presented with inactive MF, but significant fibrotic tissue damage at baseline, as shown by the absence of [^68^Ga]Ga-FAPI-04 accumulation, enhanced LGE and prolonged T1 relaxation times (Fig. 3B). On follow-up, MF became active as indicated by pronounced [^68^Ga]Ga-FAPI-04 uptake associated with increasing serum-levels of Nt-pro-BNP, new onset of atrial fibrillation, and decreased EF.

#### Early diagnosis of MF

In participant 11, pronounced [^68^Ga]Ga-FAPI-04 uptake was observed in both ventricles at baseline. At this time, cMRI did not show evidence of MF (supplementary Fig. 6A). On follow-up, the patient deteriorated clinically with declines in EF and increases in serum NT-pro-BNP. Myocardial biopsy (right ventricle) confirmed MF.

#### Imaging patterns in patients with stable disease

Participant 14 showed MF on cMRI at baseline and mild [^68^Ga]Ga-FAPI-04 uptake, consistent with low molecular activity. On follow-up, MF remained stable on cMRI and clinical parameters had improved. All follow-up investigations are visualized in Fig. 3 and supplementary Fig. 6.

## Discussion

Here, we demonstrate the feasibility of directly detecting myocardial fibroblast activation in SSc-related MF on the molecular level in vivo. As proof-of-principle, we show that [^68^Ga]Ga-FAPI-04 tracer uptake histologically corresponds to myocardial regions with accumulation of FAP^+^ myofibroblasts. Moreover, we observed that changes of [^68^Ga]Ga-FAPI-04 uptake between baseline and follow-up paralleled the changes of clinical parameters. Consistent with the pathophysiology of SSc-related MF, the distribution of myocardial [^68^Ga]Ga-FAPI-04 uptake is diffused. While [^68^Ga]Ga-FAPI-04 uptake directly visualizes activated fibroblasts, quantitative cMRI-based techniques reflect changes in the extracellular space and thus represent accrual of fibrotic damage rather than current activity. This observation is also supported by the different dynamics of change of [^68^Ga]Ga-FAPI-04 uptake and cMRI-based mapping techniques on follow-up.

Previous studies characterized FAPI uptake in other fibrotic organ changes such as the lung: In a proof-of-concept study, we recently demonstrated increased pulmonary [^68^Ga]Ga-FAPI-04 uptake in patients with SSc-related interstitial lung disease at risk of progression and that increased [^68^Ga]Ga-FAPI-04 uptake at baseline is associated with the deterioration of lung function on follow-up [16]. Other studies characterized the role of FAPI-PET CT in idiopathic pulmonary fibrosis [21, 22]. As limitation of this study, the patient number is too small to apply advanced statistical models for the analysis of the association of [^68^Ga]Ga-FAPI-04 uptake with the course of myocardial fibrosis. This is due to the situation that [^68^Ga]Ga-FAPI-04-PET-CT is a novel imaging approach and because SSc is an orphan disease with only a subset of patients developing MF. Thus, the analysis of the potential prognostic role of myocardial [^68^Ga]Ga-FAPI-04 uptake for the course of myocardial fibrosis will be subject of larger multicenter studies with higher patient numbers and longer follow-up-times.

Another limitation of the study is that the median age between the control group (51 (44.3–54.3)) and the SSc patients groups (SSc-MF patients: 59.5 (58.0–63.3), SSc-no-MF-patients: 56.5 (47.8–67)) differed. The influence of age on myocardial [^68^Ga]Ga-FAPI-04 uptake has not been investigated; however, an influence on the results cannot fully be excluded. Moreover, some limitations in spatial and temporal resolution in myocardial imaging are inherent to the PET-imaging technique: limited spatial resolution of the myocardial wall might result in underestimation of the myocardial activity concentration [23]. Temporal resolution is limited due to the relatively long PET measurement frame (2 min) compared to the dynamics of cardiac movements which can potentially be anticipated by cardiac and respiratory gating [23]. However, a gold standard has not been established yet. Despite these limitations, we observed the consistent [^68^Ga]Ga-FAPI-04 uptake in patients with myocardial fibrosis and consistent parallels between FAPI uptake and the clinical disease course.

In summary, we present first evidence that [^68^Ga]Ga-FAPI-04-PET-CT can visualize the current cardiac molecular fibroblast activity and might improve early assessment of treatment responses.

## Supplementary Information

Below is the link to the electronic supplementary material.

Supplementary file1 (DOCX 64.1 KB)

Supplementary file2 (PDF 5906 KB)

## Data Availability

All data are available upon request from the corresponding author with publication.
