# Supplementary material for: Assessment of myocardial fibrosis in patients with systemic sclerosis using [68Ga]Ga-FAPI-04-PET-CT
Source: Eur J Nucl Med Mol Imaging. 2022 Dec 16;50(6):1629–35. doi: 10.1007/s00259-022-06081-4 (PMC10119041; doi:10.1007/s00259-022-06081-4)
Supplement: Supplementary file 1 — Supplementary file1 (DOCX 64.1 KB) [file 259_2022_6081_MOESM1_ESM.docx]

**Assessment of myocardial fibrosis in patients with systemic sclerosis**

**using [^68^Ga]Ga-FAPI-04-PET-CT**

Christoph Treutlein^1^(MD), Jörg HW Distler^2,3*^(Prof), Koray Tascilar^2,3*^(MD), Sara Chenguiti Fakhouri^2,3*^(MSc), Andrea-Hermina Györfi^2,3^(MD), Armin Atzinger^4^(MD), Alexandru-Emil Matei^2,3*^(MD), Clara Dees^2,3^(PhD), Maike Büttner-Herold^5^(Prof), Torsten Kuwert^4^(Prof), Olaf Prante^4^(Prof), Tobias Bäuerle^1^(Prof), Michael Uder^1^(Prof), Georg Schett^2,3^(Prof), Christian Schmidkonz^4,6**^(Prof), Christina Bergmann^2,3**^(MD)

(*, **) These authors contributed equally

^1^Department of Radiology, Friedrich-Alexander University Erlangen-Nürnberg (FAU) and Universitätsklinikum Erlangen, Erlangen, Germany. ^2^Department of Internal Medicine 3 - Rheumatology and Immunology, FAU Erlangen-Nürnberg and Universitätsklinikum Erlangen, Erlangen, Germany; ^3^Deutsches Zentrum Immuntherapie (DZI), FAU Erlangen-Nürnberg and Universitätsklinikum Erlangen, Erlangen, Germany; ^4^Department of Nuclear Medicine, FAU Erlangen-Nürnberg and Universitätsklinikum Erlangen, Erlangen, Germany; ^5^Department of Nephropathology, Institute of Pathology, Friedrich-Alexander-Universität Erlangen-Nürnberg (FAU), Erlangen, Germany; ^6^ University of Applied Sciences Amberg-Weiden, Institute for Medical Engineering, 92637 Weiden, Germany.

*Acknowledgements:* The authors gratefully acknowledge iTheranotics (Dulles, VA, USA) for providing the precursor FAPI-04.

*Corresponding author:* Christina Bergmann*,* MD; Department of Internal Medicine 3 – Rheumatology and Immunology, Friedrich-Alexander-University Erlangen-Nuremberg (FAU) and University Hospital Erlangen, Ulmenweg 18, 91054 Erlangen, Germany, Tel.: +49 9131 43114, Email: Christina.bergmann@uk-erlangen.de, ORCID-ID: 0000-0001-5257-9171

*Materials and methods*

*Study design and participants*

In this exploratory study, six patients with Systemic Sclerosis (SSc)-related myocardial fibrosis (MF) were consecutively recruited at the Department of Internal Medicine and Rheumatology of the University Hospital Erlangen between January 2020 and December 2020. SSc-patients with SSc-related-MF based on cardiac MRI (cMRI) findings within the previous three months, fulfilling the classification criteria of the American College of Rheumatology (ACR) and European Alliance of Associations for Rheumatology (EULAR) 2013, were eligible for the study. The complete inclusion and exclusion criteria are listed in detail in supplementary table 1. Key inclusion criteria were: diagnosis of SSc according to the classification criteria of the American College of Rheumatology (ACR) and EULAR, evidence of SSc-related MF on cMRI by two independent radiologists, who were blinded for clinical information, onset of SSc (defined by the first non-Raynaud Symptom) within five years before inclusion or signs of progressive myocardial disease after five years. Exclusion criteria included: overlap disease (overlap of SSc with other connective tissue diseases), stenosing coronary artery disease, pulmonary hypertension, malignancy and previous chemotherapy.

Eight age-and sex-matched SSc-patients without signs of myocardial disease as determined clinically, by echocardiography, electrocardiogram and cMRI and six individuals without SSc and without signs of cardiac disease were consecutively recruited as controls. A participant flow diagram is shown in supplementary figure 1A. The study was performed in accordance with the Declaration of Helsinki and all procedures performed in studies involving human participants were in accordance with the ethical standards of the institutional research committee. Subjects were enrolled in the study after signing the informed consent form.

[^68^Ga]Ga-FAPI-04 is an investigational radiopharmaceutical product and is not yet approved by the Food and Drug Administration (FDA) or the European Medicines Agency (EMA) for routine medical use. [^68^Ga]Ga-FAPI-04 was therefore administered under the conditions outlined in §13(2b) of the Arzneimittelgesetz (AMG; German Medicinal Products Act), in compliance with the Declaration of Helsinki and in accordance with the ethical standards of the institutional research committee. Subjects were enrolled in the study after signing the informed consent form.

Two SSc patients with MF underwent a myocardial biopsy upon informed consent during the clinical work-up. Myocardial tissue sections were retrospectively analyzed histologically and by immunofluorescence-staining. Tissue sections from myocardial biopsies of two patients, who underwent myocardial biopsy during the clinical work-up after heart transplantation of a healthy donor heart, were retrospectively analyzed as controls. These retrospective analyses of tissue sections have also been approved by the ethics committee of the Friedrich-Alexander-University Erlangen.

*Study assessments*

The patients’ assessments during the study and pre-study phase were performed in accordance with the standards of care recommended by the European Scleroderma Trials and Research Group (EUSTAR) for SSc patients . The physicians who performed the clinical assessments were blinded to the study outcomes. A pictogram of the assessments included in the trial is shown in supplementary figure 1B. All patients underwent regular clinical examinations and investigations including serum Nt-pro-BNP, 12-lead-ECG every three months. [^68^Ga]Ga-FAPI-04-PET-CT scanning was performed at least once in all patients before inclusion into the study. In four patients, follow-up imaging with [^68^Ga]Ga-FAPI-04 PET-CT or cMRI was performed after 6 months. One patient died before follow up and another patient was not available due to change of residence. SSc-patients without signs of MF and control individuals were followed as summarized in supplementary figure 1B. Characteristics of SSc-patients and controls are summarized in supplementary table 2.

*Histological analysis*

Formalin-fixed, paraffin-embedded myocardial tissue sections were deparaffinized and stained with sirius red (Sigma-Aldrich, Steinheim, Germany) according to the manufacturers protocol for direct visualization of collagen fibers.

*Immunofluorescence*

Formalin fixed, paraffin-embedded tissue sections were deparaffinized and rehydrated. Epitope retrieval was achieved using a heat-induced method. Briefly, sections were boiled in sodium citrate buffer (10 mM sodium citrate, pH 6.0) or in Tris-EDTA buffer (10 mM Tris base, 1 mM EDTA, 0.05% Tween 20, pH 9.0). Next, sections were blocked for 1 h in phosphate-buffered saline (PBS) supplemented with 5% BSA. Primary antibodies were incubated overnight at 4 °C, after washing, secondary antibodies were incubated for 1 h at room temperature and then 4′,6-Diamidin-2-phenylindol (DAPI) staining (#3598, Santa Cruz Biotechnology Germany, Heidelberg, Germany) was performed. The following primary antibodies were used: prolyl-4-hydroxylase-β (P4Hβ, mouse mAb against P4Hβ, AF0910-1, Acris, Herfold, Germany), α-smooth muscle actin (αSMA, mouse mAb, A5228, Sigma Aldrich) and fibroblast activation protein (FAP, sheep pAB AF3715, R&D Systems, Minneapolis, Canada). Concentration-matched species-specific immunoglobulins (Vector Laboratories Germany, Eching, Germany) served as control antibodies. The staining was analyzed using a Nikon Eclipse 80i microscope (Nikon). Voronoi tessellation of *in vivo* immunofluorescence pictures were performed using the ImageJ2 software .

*Radiosynthesis and Formulation of [^68^Ga]Ga-FAPI-04*

The radiosynthesis of [^68^Ga]Ga-FAPI-04 was performed following the procedure of Linder et al.. The precursor FAPI-04 was kindly provided by Prof. Uwe Haberkorn (University Hospital Heidelberg / German Cancer Research Center (DKFZ), Germany) and by iTheranostics, Inc, a Delaware corporation (Dulles, VA, USA). The automated GMP-compliant radiosynthesis of [^68^Ga]Ga-FAPI-04 started with the elution of ^68^Ga^3+^ (5.5 mL, 0.1 M HCl) from the ^68^Ge/^68^Ga radionuclide pharmacy grade generator (1850 MBq, GalliaPharmTM, Eckert & Ziegler AG, Berlin, Germany) to a solution of FAPI-04 (30 µg in 1.5 M Hepes buffer, 3 mL) and acetic acid (0.13 mL). The reaction vial stayed at 100°C for 12 min before [^68^Ga]Ga-FAPI-04 was isolated by solid-phase extraction (Sep-Pak C18 Plus Light, 130 mg, Waters GmbH, Eschborn, Germany), elution with ethanol/water (1:1, 2 mL) and final formulation by dilution with 14ml PBS and subsequent sterile filtration. The radioactivity yield of [^68^Ga]Ga-FAPI-04 was 47±5 % at the end of synthesis. [^68^Ga]Ga-FAPI-04 was released for patient use after full quality control ensured compliance with the specification (in particular: radiochemical purity ≥ 98% (radio-TLC, radio-HPLC), FAPI-04 < 2 µg/mL (HPLC), endotoxin concentration < 17.5 EU/mL, pH = 7.5).

*[^68^Ga]Ga-FAPI-04-PET-CT-imaging procedure*

All patients were imaged on a Biograph mCT 40 PET/CT scanner (Siemens, Erlangen, Germany). Patients intravenously received an average of 1.5 MBq/kg body weight [^68^Ga]Ga-FAPI-04. At 15 minutes post injection, a nonenhanced computational tomography (CT) of the thorax was obtained with 120 kV tube voltage and with CARE Dose4D tube-current modulation (Siemens, Erlangen, Germany) set at 40mAs reference. The slice collimation of the CT was 16×1.2-mm, the rotation time and pitch were 0.5-s and 1.0, respectively. The CT-data were reconstructed with filtered back projection using B30f and B70f kernels at slice thicknesses of 1.5 and 3 mm. Following the CT, the positron-emission-tomography (PET)-scan was commenced. Attenuation-corrected PET-data were reconstructed using an OSEM algorithm and time-of-flight technology with 2 iterations/21 subsets and 6 mm full-width-at-half-maximum Gaussian post-smoothing.

*PET image analysis*

*Visual analysis*

All PET/CT datasets were analysed with commercially available software (Syngo. via, Siemens Molecular Imaging, Hoffman Estates, Illinois, USA), allowing review of PET, CT and fused imaging data. Visual interpretation of coronal, sagittal and transverse slices by two nuclear medicine specialists and one radiologist in consensus. Both nuclear medicine specialists and the radiologist were masked to clinical information. Both attenuation-corrected and non-attenuation-corrected images were reviewed visually. Myocardial tissue was considered as FAP-positive when an increased [^68^Ga]Ga-FAPI-04 uptake, higher than the nonspecific background in the blood pool (aortic vessel) was present.

*Semiquantitative analysis*

First, [^68^Ga]Ga-FAPI-04-uptake was quantified on the organ level: Therefore, mean and maximum standardized uptake value (SUVmean, SUVmax) and the metabolic active volume (MAV) of each lesion were determined manually in VOIs with isocontours set at 45% of the maximum within the respective focus, by a three dimensional segmentation and computerized volumetric technique, as performed in previous studies. Additionally, the SUVmax of the mediastinal blood pool above the aortic root was measured to calculate tissue to background ratios (TBR). From these parameters global [^68^Ga]Ga-FAPI-04 uptake was calculated by multiplying the respective SUVmean and MAV, yielding the total lesion FAPI (TL-FAPI). In addition, to describe the spatial distribution of [^68^Ga]Ga-FAPI-04-uptake in the heart, the 17 regions-model described by the American Heart association was used: This model describes 17 anatomical regions of the left ventricle (9). SUV mean and SUV max were determined according to these described anatomical areas.

*Cardiac MRI imaging protocol*

All patients were imaged on the same 1,5 T MRI scanner (Magnetom Aera, Siemens Healthineers, Erlangen, Germany) in supine position, using the same 16-channel phased array body coil. Protocol and slice planning were identical in all cases. Imaging protocols consisted at a minimum of an electrocardiography (ECG)-gated cine section, late gadolinium enhancement (LGE) imaging and pre- and post-contrast T1-mapping . Two experienced radiologists analyzed all datasets independently. Postprocessing was performed using CVI42 software (Version 5.12; Circle Cardiovascular Imaging Inc., Calgary, Canada). For analysis, the American Heart Association (AHA) 17-segment model was used .

*Volumes and Function:*

A retrospective ECG-gated segmented k-space balanced steady-state free precision (SSFP) pulse sequence with multiple breath hold commands was used (Siemens Healthineers). Three long-axis (LAX) views (four-, three-, and two-chamber views) and contiguous short-axis (SAX) views covering the entire LV from the base to the apex were acquired with in-plane spatial resolutions of 1.6 mm by 1.6 mm.

Endo- and epicardial borders of the left ventricle were semi-automatically determined on each slice in end-systolic and end-diastolic phase. Trabeculae, papillary muscles, pericardium and epicardial fat were consequently excluded from contouring. Contours rendered by automated analysis were reviewed and manually corrected, as necessary.

*T1-mapping:*

T1-mapping was performed using an ECG-gated modified Look-Locker inversion recovery (MOLLI) sequence type 5(3)3 with SSFP image readout (MyoMaps, Siemens Healthineers, Erlangen, Germany) in three short axis views (base, mid-cavity and apex) at two distinct time points within a CMR scan: once following cine imaging (pre-contrast), once 13 minutes following long axis LGE imaging (post-contrast). Basal section was defined as a fixed distance of 2cm to the mitral annulus in a diastolic four chamber view. Mid-cavity and apical sections were defined in the same four chamber view with a gap of 2 cm each.

Motion correction was performed using a variational non-rigid registration algorithm, aligning all frames to the center frame. Optimal ECG-gating and breath-holding were ensured and raw images as well as error maps were screened for potential image artifacts. In cases of suboptimal measurement, acquisition was repeated immediately.

*Late Gadolinium Enhancement:*

LGE images were acquired over slice positions matched to cines about 10 minutes following intravenous gadolinium-based contrast administration (0.02 mmol/kg Gadovist ®, Bayer, Leverkusen, Germany) with in-plan spatial resolutions of 1.8 mm by 1.3 mm and slice thicknesses of 6mm with 4mm gap. First, LAX views were acquired, SAX views followed post-contrast T1-mapping sequences. LGE images were obtained using inversion-recovery gradient echo sequences with inversion times set to null myocardial tissue signal, ranging from 240 to 300ms.

The presence of myocardial LGE, considered to reflect ECM accumulation in fibrotic tissues, was qualitatively assessed by means of visual inspection of all available LGE images, with additional quantitative analysis performed by using a signal intensity threshold of five standard deviations above visually normal remote myocardium .

*Statistical analysis*

Descriptive statistics were computed for continuous and categorical variables, which are expressed as median with interquartile range. Between-group differences were evaluated using the Mann Whitney test (in case of two groups) or Dunn´s multiple comparison test (in case of > 2 groups). P-values were considered exploratory and were not adjusted for multiple comparisons (30), a p-value < 0.05 was considered statistically significant. Statistical analyses were performed using the graph pad prism software, version 5 and R v. 4.0.1 (R Foundation for Statistical Computing, Vienna, Austria).

*Supplementary tables*

| **Inclusion criteria** | **Exclusion criteria** |
| --- | --- |
| - FAPI-PET CT before baseline - age >18 years - fulfillment of the 2013 ACR/EULAR criteria for SSc - SSc related myocardial fibrosis confirmed by cardiac MRI within 3 months before inclusion into the study as confirmed by two independent radiologists blinded for clinical information of the patients - onset of SSc (first non-Raynaud manifestation) ≤ 5 years or signs of progressive myocardial disease after five years of diagnosis | - age > 80 years - overlap disease (overlap of SSc with other CTD) - pulmonary hypertension (PH) as defined by:   - previous clinical or echocardiographic evidence of significant right heart failure   - history of right heart catheterization showing a cardiac index < 2l/min/m^2^-   - PH requiring therapy with prostacyclines (parenteral or oral, e.g. epoprostol, treprostinil, selexipag) - stenosing coronary artery disease - malignant disease - recent chemotherapy - previous hematopoietic stem cell transplantation (HSCT), or HSCT planned within the next year - pregnancy |

*Supplementary Table 1: Inclusion and exclusion criteria.* ACR: American Collegue against Rheumatism. EULAR: European Alliance of Associations for Rheumatology. SSc: Systemic Sclerosis, MRI: Magnet Resonance Imaging, CTD: connective tissue disease, PH: Pulmonary hypertension, HSCT: Hematopoetic Stem Cell Transplantation.

|  | **SSc patients,**  **MF** | **SSc patients,**  **without MF** | **non-diseased controls** |
| --- | --- | --- | --- |
| **Age (years (median, IQR))** | 59.5 (58.0-63.3) | 56.5 (47.8-67) | 51 (44.3-54.3) |
| **Gender**  Male  Female | 4/6  2/6 | 3/8  5/8 | 2/6  4/6 |
| **BMI (kg/m^2^ (median, IQR))** | 22.8 (22.3-28.4) | 24.0 (22.1-25.6) | 28.1 (26.5-29.7) |
| **Diabetes mellitus** | 1/6 | 0/8 | 1/6 |
| **Arterial hypertension** | 4/6 | 1/8 | 0/6 |
| **Chronic kidney dysfunction** | 0/6 | 0/8 | 0/6 |
| **Disease duration (since first non-Raynaud manifestation, years, median, IQR)** | 5.1 (3.5-9.0) | 4.2 (1.75-4.75) |  |
| **SSc Disease subset**  lcSSc  dcSSc | 3/6  3/6 | 4/8  4/8 |  |
| **mRSS (median, IQR)** | 20.5 (8.25-29) | 4.5 (2-9.5) |  |
| **Disease-modifying medication**  None (N/total)  MMF (N/total)  Nintedanib (N/total)  Tocilizumab (N/total)  Rituximab (N/total) | 1/6  4*/6  2*/6  0/6  0/6 | 3/8  2*/8  1*/8  2/8  1/8 |  |
| **Autoantibodies**  ANA (N/total)  Anti-Topoisomerase (N/total)  Anti Centromer (N/total)  Anti-RNAPIII (N/total)  Anti TH/T0 (N/total) | 6/6  3/6  2/6  1/6  0/6 | 8/8  3^+^/8  3**/8  3**/8  2^+^/8 |  |
| **EUSTAR activity index** (23); median (IQR) | 2.6 (1.46-3.75) | 1.3 (1.45-2.43) |  |
| **Pulmonary fibrosis** | 3/6 | 6/8 |  |

*Supplementary Table 2: Clinical data of systemic sclerosis patients and non-diseased contros at inclusion into the study***.** dcSSc: diffuse systemic sclerosis; lcSSc: limited systemic sclerosis; LGE: late gadolinium enhancement, EUSTAR: European Scleroderma Trial and Research Group; mRSS: modified Rodnan skin score; RNAPIII: ribonucleid acid polymerase III; BMI: body mass index; IQR: interquartile range. * one patient with combined treatment: MMF and nintedanib. ** one patient presented with both RNAPIII and anti-CENB. ^+^one patient presented with both anti-Topoisomerase-antibodies and anti-Th/To.


.


|  | Patient | 1 | 2 | 3 | 4 | 5 | 6 | 7 | 8 | 9 | 10 | 11 | 12 | 13 | 14 |
| --- | --- | --- | --- | --- | --- | --- | --- | --- | --- | --- | --- | --- | --- | --- | --- |
|  |  | Baseline | | | | | | | | | | | | | |
| Left Ventricle | LVEF (%) | 58.6 | 48.3 | 40.4 | 44.8 | 65.9 | 52.4 | 51.1 | 65.2 | 47.3 | 48.6 | 57.7 | 60.8 | 64.1 | 68.4 |
|  | LVEDV (ml/m²) | 85.0 | 138.0 | 75.2 | 102. | 73.4 | 122.7 | 98.4 | 63.7 | 113.4 | 85.0 | 88.5 | 73.9 | 73.6 | 79.3 |
|  | LVESV (ml/m²) | 35.1 | 71.2 | 44.7 | 56.3 | 25.0 | 58.3 | 48.0 | 22.1 | 59.7 | 43.6 | 37.4 | 28.9 | 26.4 | 25.0 |
|  | LVSV (ml/m²) | 49.9 | 66.7 | 30.4 | 45.8 | 48.4 | 64.3 | 50.3 | 41.5 | 53.6 | 41.4 | 51.0 | 44.9 | 47.2 | 54.2 |
|  | LVMyoMass  diastolic (g/m²) | 50.1 | 72.8 | 38.6 | 42.3 | 58.2 | 48.8 | 51.5 | 42.2 | 63.4 | 52.0 | 46.3 | 39.6 | 48.4 | 42.2 |
|  | LVMyoMass  systolic (g/m²) | 44.1 | 66.1 | 40.9 | 40.1 | 64.3 | 43.1 | 51.4 | 43.1 | 74.2 | 62.4 | 50.8 | 40.9 | 49.3 | 39.1 |
|  |  | Follow up | | | | | | | | | | | | | |
|  | LVEF (%) |  | 45.3 | 19.7 |  | 65.8 |  |  |  |  |  | 56.3 |  |  | 19.7 |
|  | LVEDV (ml/m²) |  | 134.3 | 68 |  | 76.1 |  |  |  |  |  | 79.6 |  |  | 68 |
|  | LVESV (ml/m²) |  | 73.3 | 55 |  | 26 |  |  |  |  |  | 34.8 |  |  | 55 |
|  | LVSV (ml/m²) |  | 60.8 | 13 |  | 50.1 |  |  |  |  |  | 44.8 |  |  | 13 |
|  | LVMyoMass  diastolic (g/m²) |  | 67.4 | 78 |  | 60.3 |  |  |  |  |  | 69.2 |  |  | 78 |
|  | LVMyoMass  systolic (g/m²) |  | 60.5 | 75 |  | 59 |  |  |  |  |  | 65.1 |  |  | 75 |
|  |  | Baseline | | | | | | | | | | | | | |
| Right Ventricle | RVEF (%) | 38.3 | 38.2 | 29.3 | 48.1 | 53.0 | 48.1 | 51.1 | 58.8 | 57.9 | 52.3 | 51.5 | 67.5 | 60.6 | 56.5 |
|  | RVEDV (ml/m²) | 183.2 | 143.8 | 144.4 | 79.5 | 85.7 | 107.2 | 104.5 | 74.4 | 124.9 | 96.4 | 94.3 | 83.4 | 86.2 | 69.7 |
|  | RVESV (ml/m²) | 113.0 | 88.8 | 102.1 | 41.2 | 40.2 | 55.6 | 51.0 | 30.6 | 52.5 | 45.9 | 45.7 | 27.0 | 33.9 | 30.3 |
|  | RVSV (ml/m²) | 70.1 | 55.0 | 42.3 | 38.2 | 45.5 | 51.5 | 53.4 | 43.7 | 72.4 | 50.5 | 48.5 | 56.3 | 52.3 | 39.4 |
|  |  | Follow up | | | | | | | | | | | | | |
|  | RVEF (%) | 0 | 34.8 | 15.7 |  | 65 |  |  |  |  |  | 49.1 |  |  | 56.4 |
|  | RVEDV (ml/m²) | 0 | 158.2 | 127 |  | 70.5 |  |  |  |  |  | 92.3 |  |  | 58.7 |
|  | RVESV (ml/m²) | 0 | 103.0 | 107.7 |  | 24.7 |  |  |  |  |  | 46.9 |  |  | 25.6 |
|  | RVSV (ml/m²) | 0 | 55.2 | 20 |  | 45.8 |  |  |  |  |  | 45.3 |  |  | 33.1 |

*Supplementary Table 3:* Baseline and follow up functional cardiac MRI parameters for left and right ventricle; LV=left ventricle, RV=right ventricle, EF= ejection fraction, EDV= enddiastolic volume, ESV= endsystolic volume, SV= stroke volume, MyoMass= myocardial mass.

**Supplementary References:**

1. Meier FM, Frommer KW, Dinser R, Walker UA, Czirjak L, et al. Update on the profile of the EUSTAR cohort: an analysis of the EULAR Scleroderma Trials and Research group database. Ann Rheum Dis. 2012;71(8):1355-60.10.1136/annrheumdis-2011-200742

2. Faisal TR, Hristozov N, Rey AD, Western TL, Pasini D. Experimental determination of Philodendron melinonii and Arabidopsis thaliana tissue microstructure and geometric modeling via finite-edge centroidal Voronoi tessellation. Physical review E, Statistical, nonlinear, and soft matter physics. 2012;86(3 Pt 1):031921.10.1103/PhysRevE.86.031921

3. Wu M, Xiao F, Johnson-Paben RM, Retterer ST, Yin X, Neeves KB. Single- and two-phase flow in microfluidic porous media analogs based on Voronoi tessellation. Lab on a chip. 2012;12(2):253-61.10.1039/c1lc20838a

4. Lindner T, Loktev A, Altmann A, Giesel F, Kratochwil C, et al. Development of quinoline-based theranostic ligands for the targeting of fibroblast activation protein. Journal of Nuclear Medicine. 2018;59(9):1415-22

5. Schmidkonz C, Rauber S, Atzinger A, Agarwal R, Götz TI, et al. Disentangling inflammatory from fibrotic disease activity by fibroblast activation protein imaging. Ann Rheum Dis. 2020;79(11):1485-91

6. Schmidkonz C, Cordes M, Goetz TI, Prante O, Kuwert T, et al. 68Ga-PSMA-11 PET/CT derived quantitative volumetric tumor parameters for classification and evaluation of therapeutic response of bone metastases in prostate cancer patients. Annals of nuclear medicine. 2019;33(10):766-75

7. Messroghli DR, Plein S, Higgins DM, Walters K, Jones TR, et al. Human myocardium: single-breath-hold MR T1 mapping with high spatial resolution—reproducibility study. Radiology. 2006;238(3):1004-12

8. Kim RJ, Wu E, Rafael A, Chen E-L, Parker MA, et al. The use of contrast-enhanced magnetic resonance imaging to identify reversible myocardial dysfunction. New England Journal of Medicine. 2000;343(20):1445-53

9. Segmentation AHAWGoM, Imaging: RfC, Cerqueira MD, Weissman NJ, Dilsizian V, et al. Standardized myocardial segmentation and nomenclature for tomographic imaging of the heart: a statement for healthcare professionals from the Cardiac Imaging Committee of the Council on Clinical Cardiology of the American Heart Association. Circulation. 2002;105(4):539-42

10. Hanneman K, Nguyen ET, Thavendiranathan P, Ward R, Greiser A, et al. Quantification of Myocardial Extracellular Volume Fraction with Cardiac MR Imaging in Thalassemia Major. Radiology. 2016;279(3):720-30.10.1148/radiol.2015150341
