# Supplementary material for: Assessment of myocardial fibrosis in patients with systemic sclerosis using [68Ga]Ga-FAPI-04-PET-CT
Source: Eur J Nucl Med Mol Imaging. 2022 Dec 16;50(6):1629–35. doi: 10.1007/s00259-022-06081-4 (PMC10119041; doi:10.1007/s00259-022-06081-4)
Supplement: Supplementary file 2 — Supplementary file2 (PDF 5906 KB) [file 259_2022_6081_MOESM2_ESM.pdf]

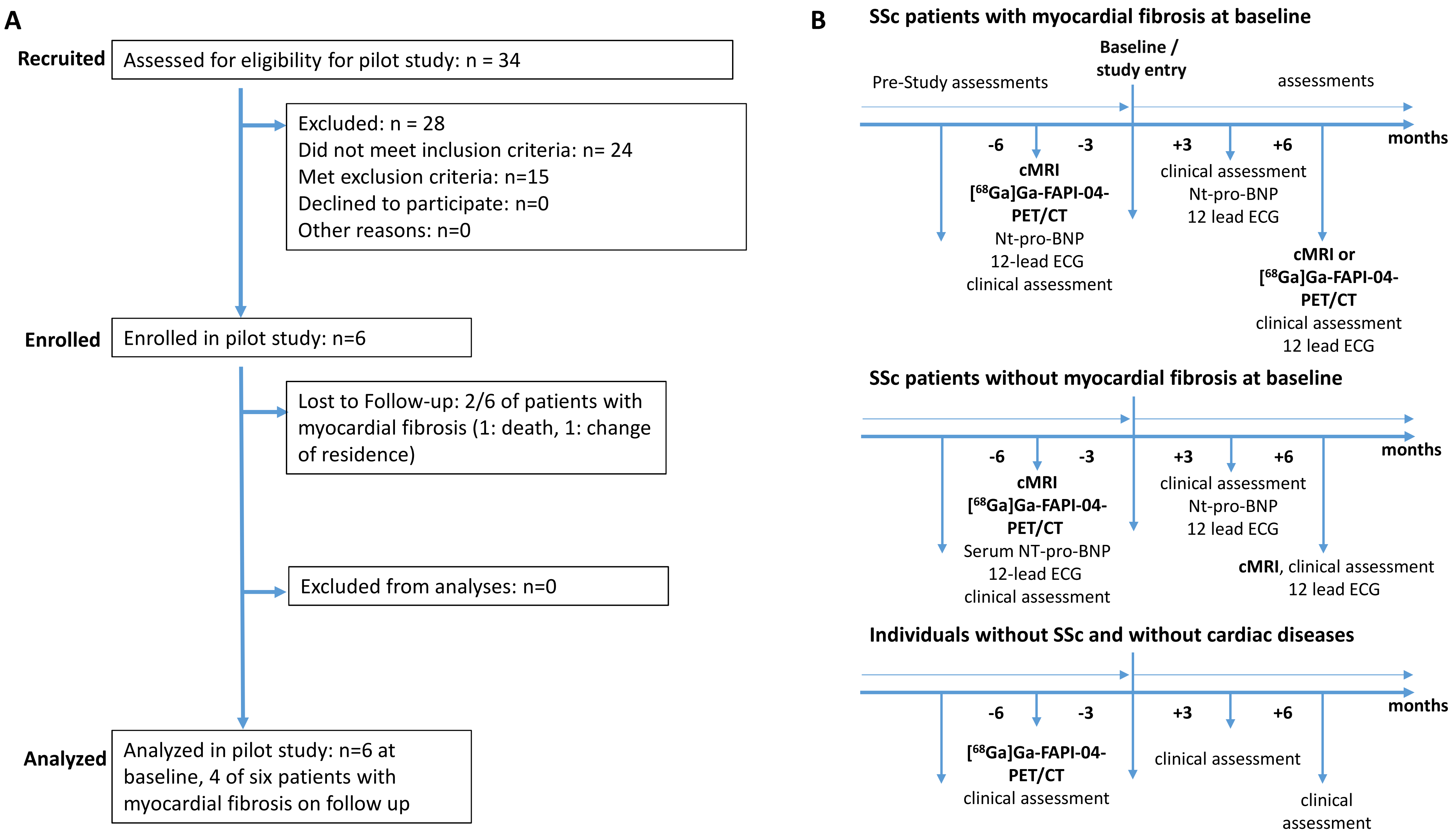

Supplementary Figure 1: A Participant flow diagram. B Pictogramm of assessments included in the trial. cMRI: cardiac MRI, ECG: electrocardiogramm, Nt-pro-BNP: N-terminal-pro- brain natriuretic peptide. [^{68}Ga]Ga-FAPI-04: ^{68}Gallium-labeled Fibroblast Activation-Protein-Inhibitor-04, PET: positron-emission tomography, CT: computational tomography

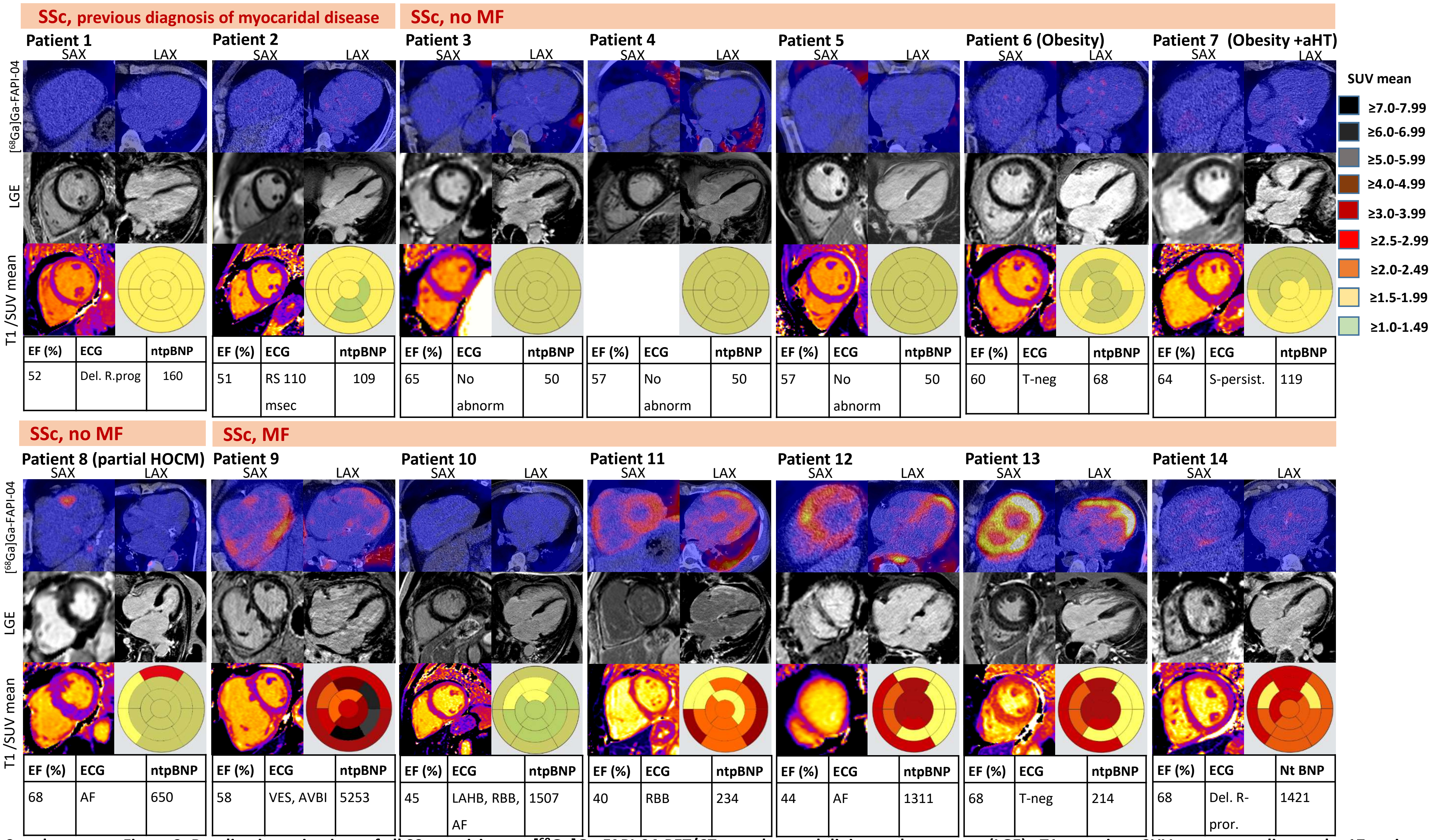

Supplementary Figure 2: Baseline investigations of all SSc-participants: [^{68}Ga]Ga-FAPI-04-PET/CT scan, late gadolinium enhancement (LGE), T1-mapping , SUV mean according to the 17-regions AHA-modell, clinical information: ejection fraction (MRI; EF; %), electrocardiogramm (ECG), nt-pro-BNP (ntpBNP, pg/ml). AHA: American heart Association, SAX: short axis-, LAX: long axis view.

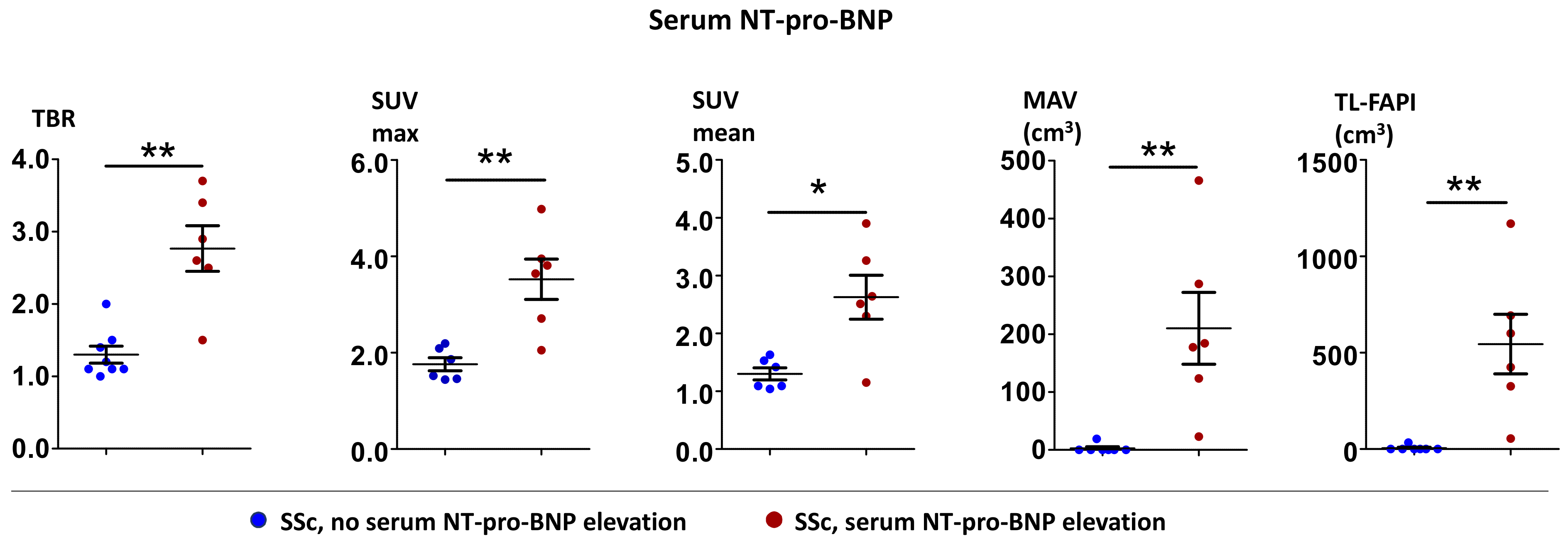

Supplementary Figure 3: [^{68}Ga]Ga-FAPI-uptake values depending on the presence of elevation of serum NT pro BNP. Results are presented as median with interquartile range. *p<0.05; **p<0.01; ***p<0.001.

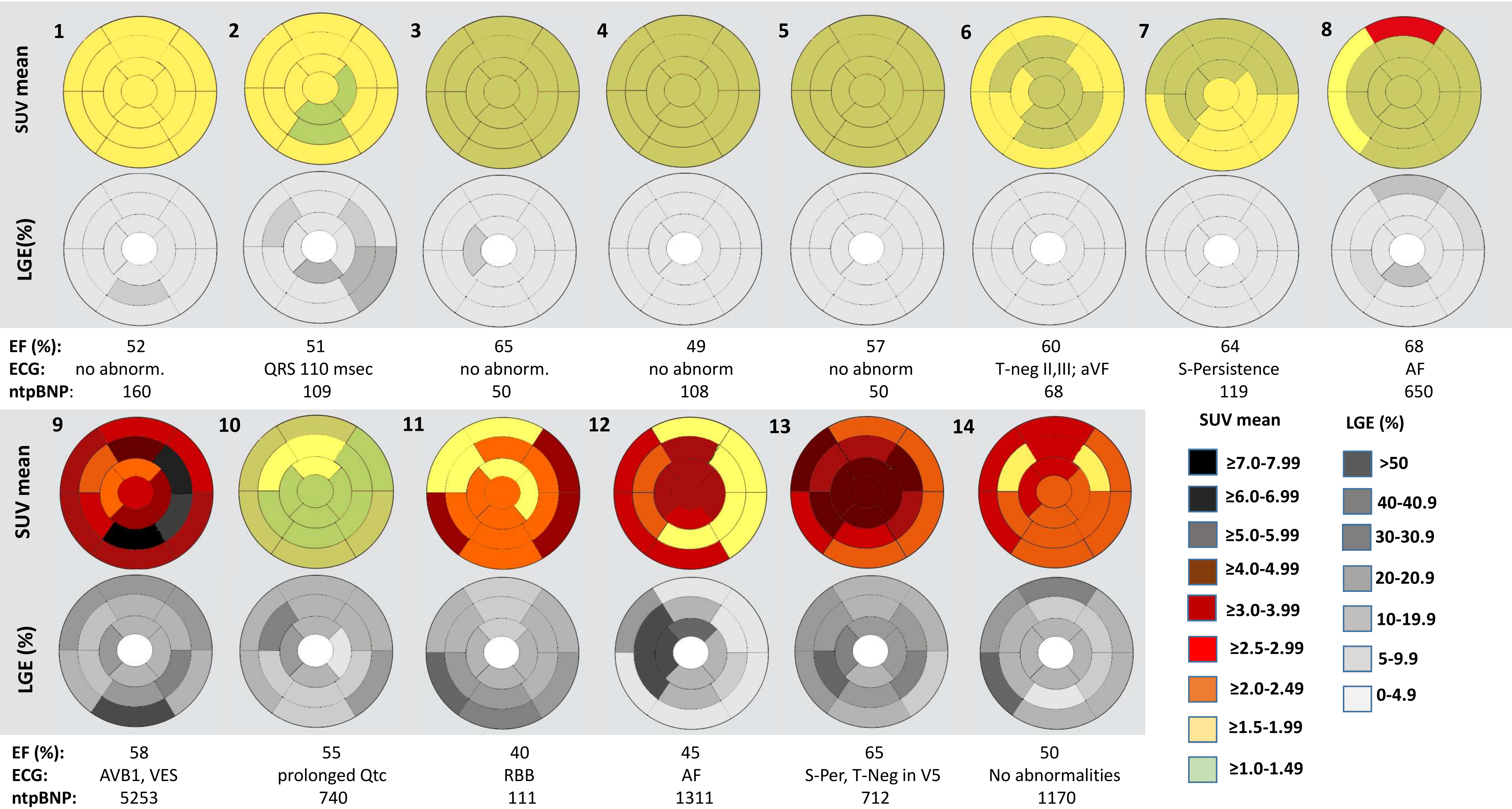

Supplementary Figure 4: Mapping of SUV mean values and LGE (%) to the 17 regions of the AHA-model in all SSc-participants (1-14) at baseline: The intensity of cMRI-based findings and ^{68}Ga-FAPI-04-uptake differ inter-individually arguing for different activity of the fibrotic remodeling process. EF: ejection fraction (%), ECG: electrocardiogram, Nt-pro-BNP (N-terminal-pro-BNP, pg/ml), AVB: atrioventricular block. VES: ventricular extrasystoles. LAHB: left anterior hemi block, RBB: right bundle block, AF: atrial fibrillation, S-Per: S-persistence, T-neg: negative T, R-progr: R-progression.

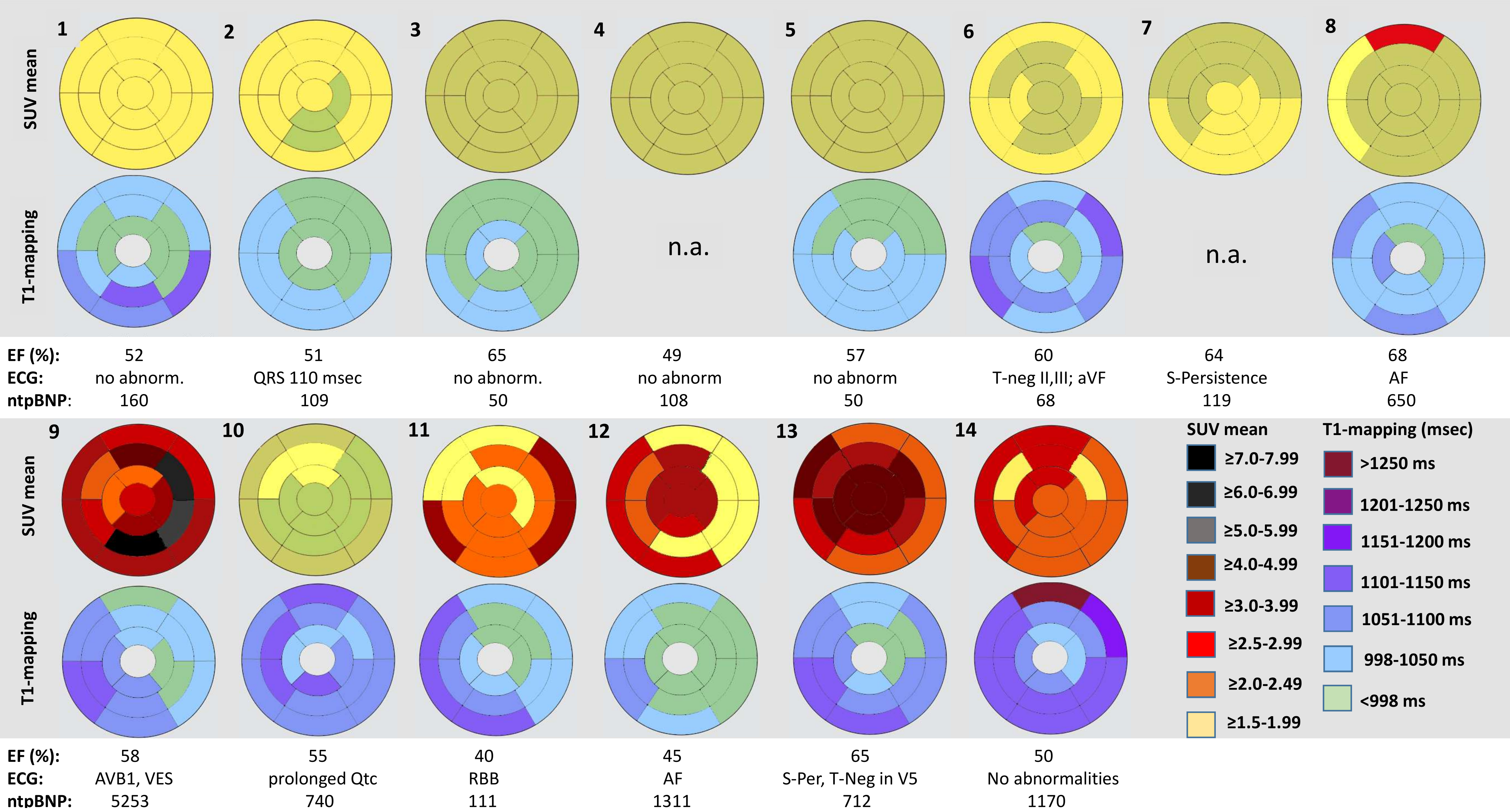

Supplementary Figure 5: Mapping of SUV mean values and T1-relaxation times to the 17 regions of the AHA-model in all SSc-participants (1-14) at baseline: The intensity of cMRI-based findings and ^{68}Ga-FAPI-04-uptake differ inter-individually arguing for different activity of the fibrotic remodeling process. EF: ejection fraction (%), ECG: electrocardiogram, Nt-pro-BNP (N-terminal-pro-BNP, pg/ml), AVB: atrioventricular block. VES: ventricular extrasystoles. LAHB: left anterior hemi block, RBB: right bundle block, AF: atrial fibrillation, S-Per: S-persistence, T-neg: negative T, R-progr: R-progression.

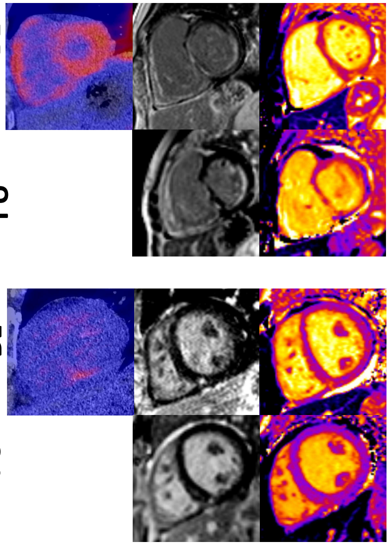

		[68Ga]Ga-FAPI-04 (MAV/TLFAP cm³)	LGE (%)	T1 relax. Time (msec)	Nt-pro-BNP (pg/ml)	EF (%)	ECG abnormalities
A	BL	SUV mean 2.1<br>MAV 286.0<br>TLFAPI 600.6	18.4	1032	111	40	RBB
	FU		20.7	1054	277	20	RBB, accumulating VES
B	BL	SUV mean 2.3<br>MAV 21.0<br>TLFAPI 54.7	15.8	1108	1170	50	No abnormalities
	FU		16.0	1041	301	57	No abnormalities

Supplementary Figure 6: **Follow up observations of participants 11 and 14**. Representative images of [^{68}Ga]-FAPI-04-PET/CT scan and the corresponding cMRI (LGE and T1) at baseline (BL) and the corresponding sections of the same modalities on follow up (FU) are shown. Clinical findings including serum Nt-pro-BNP levels, EF(%) and ECG findings are tabulated. **A**: Clinical progress on follow up despite therapy with nintedanib and MMF (Participant 11). **B:** Myocardial fibrosis on MRI and low ^{68}Ga-FAPI-04 uptake at BL and stable MRI and improved clinical parameters on FU (Participant 14). BL: baseline, FU: follow up, VES: ventricular extrasystoles, RBB: right bundle branch block, EF: ejection fraction.
